# Blood pressure response to an exercise treadmill test, and echocardiographic left ventricular geometry in Nigerian normotensive diabetics

**Published:** 2010-04

**Authors:** EA Ajayi, MO Balogun, OA Akintomide, RA Adebayo, OE Ajayi, RT Ikem, SA Ogunyemi, AT Oyedeji

**Affiliations:** Department of Internal Medicine, University Teaching Hospital, Ado Ekiti, Nigeria; Department of Medicine, Obafemi Awolowo University Teaching Hospital, Ile Ife, Nigeria; Department of Medicine, Obafemi Awolowo University Teaching Hospital, Ile Ife, Nigeria; Department of Medicine, Obafemi Awolowo University Teaching Hospital, Ile Ife, Nigeria; Department of Medicine, Obafemi Awolowo University Teaching Hospital, Ile Ife, Nigeria; Department of Medicine, Obafemi Awolowo University Teaching Hospital, Ile Ife, Nigeria; Department of Medicine, Obafemi Awolowo University Teaching Hospital, Ile Ife, Nigeria; Department of Medicine, Obafemi Awolowo University Teaching Hospital, Ile Ife, Nigeria

**Keywords:** diabetes, exercise, blood pressure response, left ventricular geometry

## Abstract

**Objectives:**

This study evaluated normotensive diabetic patients’ blood pressure response to graded exercise and their echocardiographic pattern of left ventricular geometry.

**Methods:**

A descriptive, cross-sectional, hospital-based study was carried out on 30 normotensive type 2 diabetic patients and 34 controls, aged 30 to 60 years. The outcome measures were to determine the exercise-related variable, blood pressure response, and left ventricular geometry by means of echocardiography.

**Results:**

Nineteen (29.7%) and 11 (17.2%) normotensive diabetic subjects had normal left ventricular geometry and concentric left ventricular remodelling, respectively. None of the subjects had concentric or eccentric left ventricular hypertrophy. On this basis, the normotensive diabetic subjects were divided to two groups: G1 (normal) and G2 (concentric left ventricular remodelling). The groups had comparable mean age, body mass index (BMI), fasting blood glucose (FBG) and two-hour post-prandial blood glucose values, and heart rate, systolic (SBP) and diastolic blood pressure (DBP) at rest. G2 patients had higher mean duration of diabetes than G1 subjects (69.0 ± 9.48 vs 18.7 ± 8.7 months; *p* = 0.007). Peak systolic blood pressure was significantly higher in G2 than G1 subjects (213.6 ± 20.1 vs 200.0 ± 15.3 mmHg; *p* = 0.04). Although there was no statistically significant difference in the left ventricular (LV) mass index between the groups, G2 patients had significantly higher relative wall thicknesses than G1 patients (0.53 ± 0.03 vs 0.41 ± 0.04; *p* < 0.001).

**Conclusion:**

Normotensive diabetic subjects with concentric left ventricular remodelling have increased blood pressure reactivity to exercise. It is probable, as suggested in earlier studies, that increased blood pressure reactivity to exercise is an indicator of target-organ damage, particularly in normotensive diabetics.

## Summary

Stress increases blood pressure, and variable individual blood pressure responses have been evaluated with regard to prediction of new-onset hypertension, target-organ damage and incident cardiovascular disease or death.^1^ The significance of blood pressure reactivity to exercise has been evaluated, with variable results, in studies on the association between the blood pressure response to exercise and either left ventricular mass or left ventricular geometry in hypertensive patients.^2^,^3^ The exaggerated exercise blood pressure (BP) values in these hypertensive adults have been attributed to impaired endothelial vasodilator function.^4^

Arterial stiffness is also related to type 2 diabetes,^5^ mainly due to an impaired endothelial vasodilator function, which in turn is associated with increased afterload,^5^ leading to an elevated systolic blood pressure (SBP).^6^ These processes consequently lead to structural alterations in the diabetic heart. In normotensive diabetic patients, early and asymptomatic functional and structural abnormalities may alter the normal response to exercise, as already observed in elderly^7^ and non-diabetic hypertensive patients.^3^ However, not much is known about the relationship between blood pressure response to exercise and sub-clinical cardiac end-organ damage in normotensive diabetics, particularly in Nigeria.

In light of the above, we set out to investigate the relationship between blood pressure response to graded exercise in normotensive diabetics and their echocardiographic pattern of left ventricular geometry, as evidence of cardiac end-organ damage.

## Methods

Thirty normotensive type 2 diabetic subjects (male = 15; female = 15) and 34 normal controls (male = 17; female = 17) aged 30 to 60 years were recruited through the medical out-patient department of Obafemi Awolowo University Teaching Hospitals complex (OAUTHC), Ile Ife, Nigeria. Ethical clearance for the study was approved by the Ethics and Research Committee of the Hospital in conformity with ethical guidelines of the 1975 Declaration of Helsinki, and all participants gave written consent to participate.

Demographic parameters of the subjects were noted and recorded. All subjects were clinically examined to evaluate their body mass index (BMI) and cardiovascular status at rest. Subjects were considered diabetic if they had fasting plasma glucose (FBG) values ≥ 126 mg/dl (7.0 mmol/l)^8^ or if they used hypoglycaemia medication. Fasting plasma glucose and two-hour post-prandial plasma glucose (2HPP) values were obtained 24 hours prior to the procedures.

A resting 12-lead ECG was done to exclude patients with baseline ST-segment abnormalities and bundle branch block. Also excluded were patients with coexisting hypertension or who were on antihypertensive(s), those with established chronic renal failure or serum creatinine levels > 1.5 mg% (132 μmol/l), congestive heart failure, valvular heart disease and other diseases known to influence LV function, such as thyroid disease and severe obesity.

All the subjects underwent treadmill-symptom limited maximal exercise using the Bruce protocol.^9^ The protocol continued until one of several endpoints was reached. These included if the patient achieved the age-predicted maximum heart rate; requested that the exercise be terminated; developed severe chest pain, fatigue, leg discomfort or dyspnea; developed frequent premature ventricular beats; developed a systolic blood pressure > 250 mmHg or a drop in the pre-test systolic blood pressure > 10 mmHg; or developed any other problems necessitating termination of exercise.

The subjects also had transthoracic two-dimensional (2D) and 2D derived M-mode echocardiography performed, according to standard procedure,^10^ with simultaneous electrocardiographic recordings while in the left lateral decubitus position, using a standard ultrasound machine (Sonoline G60S Ultrasound Imaging System) with 4.2-MHz transducer. Left ventricular enddiastolic measurements were taken during at least three cycles11 and included left ventricular internal diameter (LVIDD), posterior wall thickness (PWT) and interventricular septal thickness (IVST). Left ventricular mass was estimated from the American Society of Echocardiography’s formula^11^:

Estimated LV mass index (g/m^2^) = 0.80 [1.04 (LVIDD + PWT + IVST)^3^ – (LVIDD)^3^] + 0.6 g/BSA

Upper normal limits for LV mass index were 134 and 110 g/m^2^ in men and women, respectively.^12^ Relative wall thickness (2 × posterior wall thickness/LV diastolic diameter) was calculated.^13^ A partition value of 0.45 for relative wall thickness was used for both men and women.^14^ Patients with increased LV mass index and increased relative wall thickness were considered to have concentric hypertrophy, and those with increased LV mass index and normal relative wall thickness were considered to have eccentric hypertrophy. Those with normal LV mass index and increased or normal relative wall thickness were considered to have concentric remodelling or normal geometry, respectively.

## Results

The diabetic subjects and controls had comparable ages and BMIs (48.37 ± 6.96 vs 48.35 ± 6.13 years; *p* = 0.197 and 24.82 ± 3.66 vs 24.38 ± 1.94 kg/m^2^; *p* = 0.861, respectively). Diabetic subjects had significantly higher FBG values than the controls (8.94 ± 2.13 vs 4.75 ± 0.37 mmol/l; *p* ≤ 0.001).

As shown in Table 1, normotensive diabetic subjects had higher exercise-induced haemodynamic parameters of peak systolic (pSBP) and peak diastolic blood pressure (pDBP) but lower peak heart rates (pHR). There was no statistically significant difference in left ventricular mass index (LVMI). Nineteen (29.7%) and 11 (17.2%) normotensive diabetic subjects had normal left ventricular geometry and concentric left ventricular remodelling, respectively. None of the normotensive diabetic subjects had concentric or eccentric left ventricular hypertrophy. Thirty (46.8%) and four (6.3%) controls had normal left ventricular geometry and concentric left ventricular remodelling, respectively. None of the subjects had concentric or eccentric left ventricular hypertrophy.

**Table 1.** Haemodynamic Response And Echocardiographic Pattern Of The Study Population

*Parameters*	*Normotensive diabetics (n = 30)*	*Controls (n = 34)*	p*-value (Student’s t-test)*
rHR (per min)	91.37 ± 16.10	83.29 ± 5.36	0.038
rDBP (mmHg)	73.03 ± 5.46	71.94 ± 3.13	0.713
rSBP (mmHg)	117.13 ± 6.36	113.62 ± 4.51	0.044
pHR (per min)	166.00 ± 15.61	179.03 ± 9.10	< 0.001
pDBP (mmHg)	95.67 ± 9.35	89.12 ± 7.12	< 0.001
pSBP (mmHg)	205.00 ± 18.15	185.41 ± 10.81	< 0.001
Exercise capacity (METs)	8.07 ± 1.47	8.11 ± 0.88	0.992
LVMI (g/m^2^)	93.97 ± 17.04	90.55 ± 17.09	0.512
IVST (mm)	10.24 ± 1.36	9.45 ± 1.44	0.084
PWT (mm)	9.70 ± 1.51	9.43 ± 1.50	0.771
RWT	0.45 ± 0.68	0.41 ± 0.07	0.038

Statistical significance at *p* < 0.05; Values are expressed as mean ± SD; rHR = resting heart rate, pHR = peak heart rate.

The normotensive diabetic subjects were then divided into two groups: G1 (normal) and G2 (concentric left ventricular remodelling) on this basis. The groups had comparable mean ages, BMIs, FBG and two-hour post-prandial blood glucose values, heart rates, and SBP and DBP at rest (Table 2). G2 patients had a higher mean duration of diabetes than G1 (69.0 ± 9.48 vs 18.7 ± 8.7 months; *p* = 0.007). The patients’ characteristics at rest were not statistically significantly different (Table 2).

**Table 2.** Clinical And Demographic Pattern Of G1 And G2 Subjects

*Parameters*	*Normal LV geometry (n = 19)*	*Concentric LV remodelling (n = 11)*	p*-value (Student’s t-test)*
Age	48.68 ± 7.7	47.82 ± 5.7	0.749
Gender			
M: *n* (%)	7 (36.8%)	8 (72.7%)	0.058*
F: *n* (%)	12 (63.2%)	3 (27.3%)	
BMI (kg/m^2^)	24.8 ± 4.1	24.8 ± 2.9	0.992
Duration of diabetes (months)	18.7 ± 8.7	69.0 ± 9.48	0.007
FBG (mmol/l)	9.8 ± 2.03	8.1 ± 1.9	0.082
2HPP (mmol/l)	12.2 ± 1.9	13.8 ± 3.5	0.236
rHR (bpm)	92.1 ± 18.2	90.1 ± 12.4	0.748
rDBP (mmHg)	72.4 ± 5.8	74.2 ± 4.9	0.390
rSBP (mmHg)	118.5 ± 6.5	114.7 ± 5.6	0.116
rPP (mmHg)	46.2 ± 8.7	40.6 ± 3.9	0.052

Statistical significance at *p* < 0.05; *Chi-square. Values are expressed as mean ± SD.

As shown in Table 3, peak systolic blood pressure was significantly higher in G2 subjects than in G1 (213.6 ± 20.1 vs 200.0 ± 15.3 mmHg; *p* = 0.04). The difference between resting systolic and peak systolic blood pressure (ΔSBP) as well as resting pulse pressure and pulse pressure during exercise (ΔPP) followed a similar trend to that of peak systolic blood pressure. Exercise capacity in G2 subjects was significantly lower than in G1 by 12.94% (7.4 ± 1.1 vs 8.5 ± 1.5 METs; *p* = 0.042). Although, there was no statistically significant difference between the LV mass index in the two groups, G2 subjects had significantly higher relative wall thicknesses than those in G1 (0.53 ± 0.03 vs 0.41 ± 0.04; *p* < 0.001) (Table 4).

**Table 3.** Exercise-Induced Haemodynamic Factors

*Parameters*	*Normal LV geometry (n = 19)*	*Concentric LV remodelling (n = 11)*	*p-value (Student’s t-test)*
pHR (bpm)	167.8 ± 10.9	162.8 ± 21.7	0.405
pDBP (mmHg)	94.2 ± 7.7	98.2 ± 11.7	0.270
pSBP (mmHg)	200.0 ± 15.3	213.6 ± 20.1	0.045
ΔHR (bpm)	75.7 ± 18.4	72.7 ± 28.1	0.725
ΔDBP (mmHg)	21.5 ± 14.1	24.0 ± 13.3	0.596
ΔSBP (mmHg)	81.5 ± 14.1	98.9 ± 20.1	0.010
ΔPP (mmHg)	105.8 ± 9.6	115.5 ± 11.3	0.019
HR reserve	0.97 ± 0.16	0.87 ± 0.03	0.222
Exercise capacity (METs)	8.5 ± 1.5	7.4 ± 1.1	0.042

Statistical significance at *p* < 0.05 Values are expressed as mean ± SD.

**Table 4.** Echocardiographic Parameters Of G1 And G2 Subjects

*Parameters*	*Normal LV geometry (n = 19)*	*Concentric LV remodelling (n = 11)*	p*-value (Student’s t-test)*
LVMI (g/m^2^)	81.1 ± 13.4	88.9 ± 21.8	0.233
IVST (mm)	9.8 ± 1.2	11.1 ± 1.3	0.010
PWT (mm)	9.0 ± 1.3	10.9 ± 1.1	< 0.001
RWT	0.41 ± 0.04	0.53 ± 0.03	< 0.001

Statistical significance at *p* < 0.05 Values are expressed as mean ± SD.

## Discussion

The relationship of blood pressure response to exercise and endorgan damage in hypertensive subjects is not clear. Studies on this subject in diabetics are few, especially among blacks, who unfortunately are at higher risk of developing cardiovascular-related complications than their Caucasian counterparts.^15^ This study is the first in Nigeria to assess the relationship between blood pressure response to exercise and abnormal LV geometry.

In this study, gender, age and BMI were comparable among the patients with normal LV geometry and those with LV concentric remodelling. The longer duration of diabetes in patients with concentric LV remodelling supports the earlier assertion that the longer the duration of diabetes, the more the likelihood that the patient will develop cardiovascular complications. This was despite the fact that short-term (FBG, two-hour post-prandial blood glucose) glycaemic control was similar in both groups in this study, suggesting that blood pressure response during exercise may not have been much influenced by blood glucose exposure.

It has been suggested that blood pressure response may be related to blood glucose control.^16^ Marfella *et al.* reported that in the resting state, the presence of hyperglycaemia led to an increase in SBP and DBP independently of endogenous insulin in 20 patients with type 2 diabetes. A reduced availability of nitric oxide was suggested as a possible explanation.^16^

In our study, the peak systolic blood pressure during exercise was significantly higher in patients with LV concentric remodelling than in those with normal LV geometry. This however was not the case with peak diastolic blood pressure. This was reflected in the significant change in pulse pressure (ΔPP) observed during exercise. Pulse pressure provides a crude guide to stiffness of the large conduit arteries.^17^ Physiological parameters related to blood pressure regulation and potential contributors to reduced exercise capacity in type 2 diabetic individuals include reduced LV systolic volume, altered myocardial and diastolic functions and increased arterial stiffness.^5^,^18^ The elevated peak exercise SBP observed in patients with concentric left ventricular remodelling in this study was probably partly associated with arterial stiffness, as reflected by the higher ΔPP.^5^,^6^

Exercise capacity was also reduced in patients with LV concentric hypertrophy in our study. This may provide additional explanation for reduced exercise tolerance in normotensive diabetes patients. It has been suggested that the voltage on the ECG of left ventricular hypertrophy may be an early marker of impaired exercise capacity.^19^ Previous studies have shown that left ventricular hypertrophy independently predicted reduced exercise capacity.^20^ This study has shown that type 2 diabetic patients with increased peak systolic blood pressure had increased arterial stiffness, higher LVMI, abnormal LV geometry and reduced exercise capacity.

## Conclusion

Normotensive diabetics with concentric left ventricular remodelling have increased systolic blood pressure reactivity to exercise. It is probable, as suggested in earlier studies, that increased blood pressure reactivity to exercise is an indicator of target-organ damage, especially in normotensive diabetics.
